# Synchronization in flickering of three-coupled candle flames

**DOI:** 10.1038/srep36145

**Published:** 2016-10-26

**Authors:** Keiko Okamoto, Akifumi Kijima, Yoshitaka Umeno, Hiroyuki Shima

**Affiliations:** 1 Department of Environmental Sciences, University of Yamanashi, 4-4-37 Takeda, Kofu, Yamanashi 400-8510 Japan; 2 Faculty of Education, University of Yamanashi, 4-4-37 Takeda, Kofu, Yamanashi 400-8510 Japan; 3 Institute of Industrial Science, The University of Tokyo. 4-6-1 Komaba, Meguro-ku, Tokyo 153-8505 Japan

## Abstract

When two or more candle flames are fused by approaching them together, the resulting large flame often exhibits flickering, *i.e.,* prolonged high-frequency oscillation in its size and luminance. In the present work, we investigate the collective behaviour of three-coupled candle flame oscillators in a triangular arrangement. The system showed four distinct types of syncronised modes as a consequence of spontaneous symmetry breaking. The modes obtained include the in-phase mode, the partial in-phase mode, the rotation mode, and an anomalous one called the “death” mode that causes a sudden stop of the flame oscillation followed by self-sustained stable combustion. We also clarified the correlation between the inter-flame distance and the frequency with which the modes occur.

Flickering of a candle flame has been familiar to us since the invention of the candle and its subsequent use for lighting purposes. It is easily observed when you bundle a few candles, which are commercially available, and then ignite them together. If the wicks of the candles involved in the bundle are close enough to each other, then the adjacent flames merge and the resulting large flame will oscillate in the luminous intensity and geometric size with ca. 10 Hz^1^,^2^,^3^. Once getting started, the oscillation becomes self-sustained; for example, even when you breathe on the flame and stop the oscillation transiently, the oscillation restarts after the perturbation is removed. This self-sustaining oscillation in the candle flame is in a complete contrast with the stable combustion that a single, isolated candle flame exhibits.

A further interesting phenomenon associated with candle flame flickering is the spontaneous synchronisation observed in pairs^1^,^3^,^4^ and arrays^3^ of oscillating candle flames. It was found in ref. 1 that a pair of oscillating candle flames shows two distinct classes of synchronisation modes, depending on the distance between the two flames. When the distance is small enough, they exhibit in-phase synchronisation, in which both flames oscillate identically with no phase difference. When the distance is larger (but smaller than an upper limit), they exhibit anti-phase synchronisation, in which the waveforms of the two flames’ oscillations are identical but they are phase-shifted by half a period.

The argument mentioned above poses a simple question: What do we observe when three candle flames are coupled? In general, three-coupled oscillator systems are the simplest ones capable of exhibiting frustration. Frustration makes it impossible that every pairs chosen from the three-coupled oscillators goes into anti-phase simultaneously because, when two pairs of the three synchronise in the anti-phase mode, respectively, the remaining third pair must synchronise in the in-phase mode. Nevertheless, frustration plays an important role in the dynamics of three-coupled oscillators^5^, because it may cause symmetry breaking that drives certain asymmetric synchronised modes^6^. Earlier studies have demonstrated that several non-trivial mode patterns were observed in three-coupled oscillators of physical^7^,^8^,^9^,^10^, chemical^11^, and biological systems^12^,^13^,^14^ as well as human group dynamics^15^. Since the symmetry-based understanding is model independent, it is expected to hold for synchronisation of three-coupled candle flames. It was found in ref. 3 that a triangular array composed of three individual candles (not three bundles) showed an intermittent occurrence of synchronisation, though the possible types of synchronised modes that are observable in the system was not thoroughly examined.

In the present work, we investigated the synchronised oscillations of three-coupled candle flames, wherein three oscillating flames are positioned at the vertices of an equilateral triangle. Through image analysis of the three-coupled flame dynamics, we examined the kind of synchronised modes observable in the system and their consistency with the symmetry-based understanding. We also studied the relationship between the inter-flame distance (*i.e.,* coupling strength of the flames) and the most frequently occurring synchronisation mode.

## Methods

We used candles made of paraffin, obtained from a grocery store in Japan, having a thin cylindrical shape with 9.0 mm in diameter and 110 mm in height. The length of the candle wick is nearly 10 mm, with slight individual difference. To make an oscillator, we bundled three of the thin candles in parallel with tape; as a consequence, three wicks come close enough to each other so that the three individual flames merge at ignition. Next we painted side surfaces of every bundle in black for convenience in our image analysis. Afterwards, these three bundles, each of which consists of three candles, are erected vertically on a table and equi-separated; the tips of the three bundles are positioned at the vertices of an equilateral triangle.

**Figure 1:**
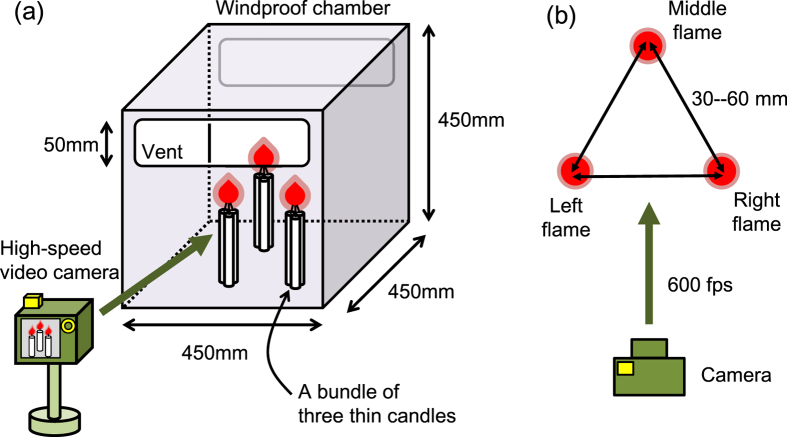
Schematic illustration of the experimental setup. (**a**) Overview. (**b**) Top view.

Figure 1 shows a schematic representation of the experimental setup. The distance between the tips of the bundles, designated by 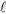, was tuned within the range of 30 mm–60 mm with keeping the equilateral triangular arrangement. To remove the disturbance from external air flow, the set of bundles was enclosed in a windproof chamber with a 450 mm cubic shape. The chamber is made of transparent acrylic, having two vents with 50 mm in height at the front and back faces of the chamber’s upper end. It was confirmed that the oscillation behaviours of the candle flames were independent of the presence/absence of the chamber, as attributed to that the length of the side of the chamber is sufficiently larger than the flame-to-flame distance.

The dynamics of three-coupled flame combustion was recorded under a dark background using a high-speed video camera (EXILIM EX-F1, CASIO, Japan) at 600 frames per second. A two-minute-long movie was recorded in grayscale per experimental trial, and then it was analysed to pursue the time variation in the oscillating flame sizes. The flame size at a certain moment was estimated by counting up the “bright” pixels in an adequate area on every frame of the movie. Given a grayscale image, in general, the brightness of each pixel is represented by an 8-bit integer ranging from 0 (pure black) to 255 (pure white). Among 256 different intensities, those greater than 200 were recognized as “bright”.

## Synchronisation modes of three-coupled candle flames

The three-coupled candle flames turned out to exhibit four classes of synchronised oscillation modes. Essential features of the four modes are explained below in turn.

### In-phase mode

**Figure 2:**
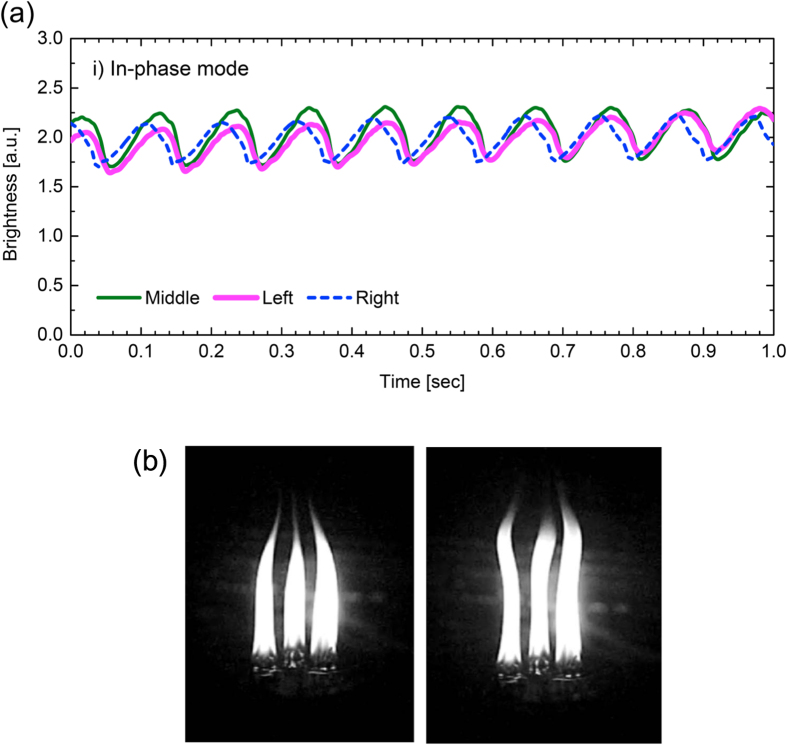
In-phase synchronisation mode of the three-coupled candle flames. All the three oscillators obey an identical waveform. (**a**) Time series of the flame brightness. (**b**) Snapshot of the oscillating flames.

The first synchronisation to be mentioned is the in-phase mode, in which all the three flames oscillate with an identical wave form. Figure 2(a) demonstrates the flame dynamics of the in-phase mode in a one-second duration. This time series was obtained by an experiment with the inter-flame distance of 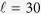 mm; A one-second portion was then extracted randomly from the two-minute movie. The graph indicates that, in this one-second duration, all the three flames oscillate with nearly the same amplitude and frequency ~10 Hz. The mean value deduced from many trials on the in-phase mode was 10.1 Hz with the standard deviation of ±0.5 Hz. Figure 2(b) shows a snapshot of the oscillating flames in the in-phase mode. During the oscillation, the visible parts of the flames are elongated and contracted periodically in the vertical direction. In addition, a transverse oscillation was observed; bowing to the center of the triangle (left panel) and arching backward (right panel) were repeated in the same frequency as that of the vertical oscillation. A footage can be found in the Supplementary Video S1. This arching/bowing phenomenon was found to occur in *n*-coupled candle flames with *n* ≥ 3 in the work done by ref. 3.

It should be noted that the in-phase mode observed in three-coupled candle flames is unstable. The sustained time duration is only a few seconds at most, even when the system is free from external air flow. Moreover, the degree of synchronisation dissipates as time passes, followed by the occurrence of another synchronisation mode or the complete disappearance of synchronisation. This instability is in contrast with the stable oscillation in a single and paired candle flames^1^. It is interesting to compare Supplementary Video S1 in the present work with Supplementary Information S2 in ref. 3, which demonstrates the stable dynamics of the paired flame’s in-phase mode.

Another important feature of the time series variation shown in Fig. 2 is the geometric asymmetry in the waveform. It is observed that all the three waveforms deviate significantly from a sinusoidal wave. That is, the waveform for a single period is slanted; the curve shows a gentle incline from a minimum (*e.g.*, at *t* = 0.06 sec) to the immediately following maximum (*t* = 0.12 sec), followed by a steep decline from the maximum to the subsequent minimum (*t* = 0.16 sec). The asymmetry in the waveform indicates the fact that a little larger time duration is required for the flames to complete a single period of elongation, compared with the time duration required for them to complete a single period of contraction.

### Partial in-phase mode

**Figure 3:**
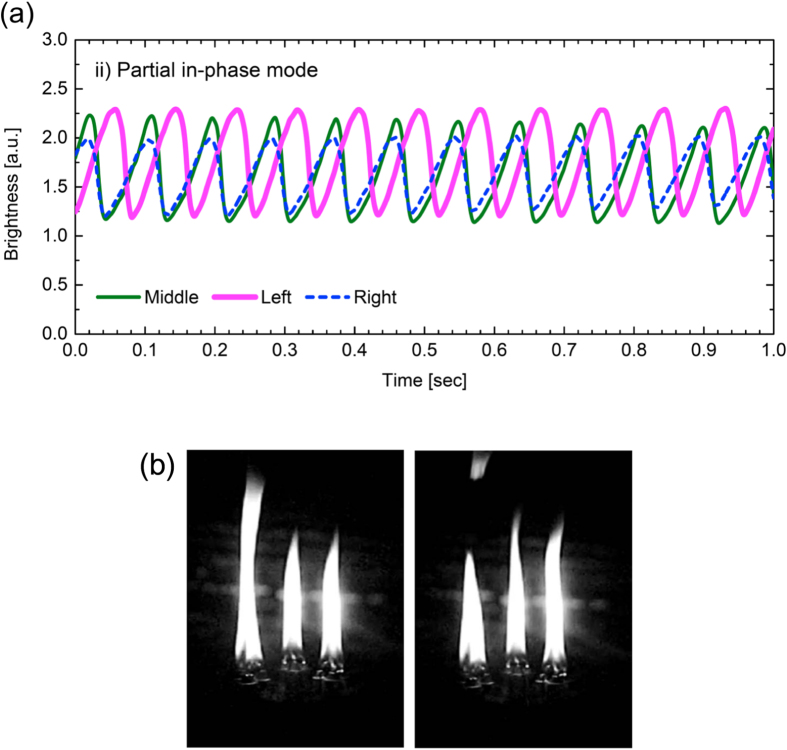
Partial in-phase mode. Two oscillators are in-phase synchronisation, while the remaining one is phase-shifted by *π*. (**a**) Time series of the flame brightness. (**b**) Snapshot of the oscillating flames.

The second synchronisation mode is the partial in-phase mode. In this mode, two of three oscillators have nearly the same waveform and are in-phase, but the remaining one oscillator is phase-shifted by *π*. Figure 3(a) represents the time variation in the flame sizes when the inter-flame distance was set to be 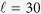 mm. The result indicates that the middle and right flames oscillate in nearly the same timing but the left flame oscillates with a delay of half a period. The snapshot presented in Fig. 3(b) will be of help to capture the dynamics of the three-coupled flames. We have observed in experiments that, when the partial in-phase mode occurs, there seems to be no preference of a pair among the three oscillators to become in-phase coupled over the other two pairs. The pair was chosen stochastically and thus changed every experimental trial. Similarly to the case of the in-phase mode, the partial in-phase mode is also unstable and endowed with asymmetry in the waveform. The oscillation frequency is estimated to be of 11.5 ± 0.3 Hz a bit larger than that for the in-phase mode explained in the previous subsection.

### Rotation mode

**Figure 4:**
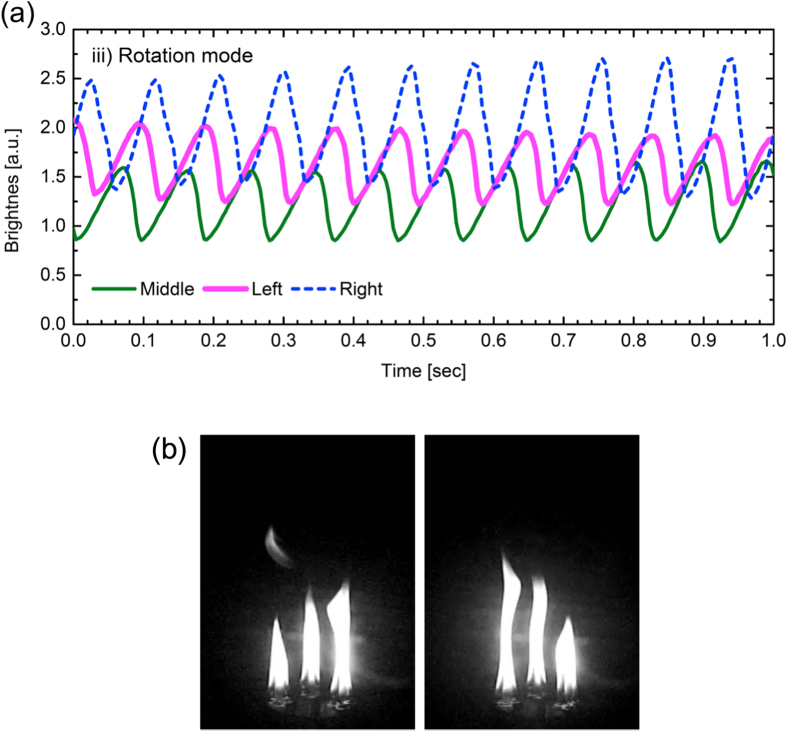
Rotation mode. All three oscillators are synchronised with a constant phase difference of *π*/3. (**a**) Time series of the flame brightness. (**b**) Snapshot of the oscillating flames.

The third to be noted is the rotation mode, wherein all three oscillators are synchronised with a constant phase difference of *π*/3. Figure 4 shows the flame dynamics of the rotation mode obtained for 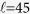 mm. The three flames repeat elongation and contraction in an alternate manner; as a result, the collective oscillation of the flames seems visually analogous to the “Mexican wave” that is often raised by spectators in the sport stadium. The alternately oscillating behaviour is visually confirmed by observing the footage in Supplementary Video S4. The rotative direction, clockwise or counterclockwise, was randomly chosen, as was confirmed by experiments. The oscillation frequency is estimated to be of 11.0 ± 0.4 Hz. Again, the instability of synchronisation and asymmetry in the waveform were both observed in the rotation mode, too.

### Death mode

**Figure 5:**
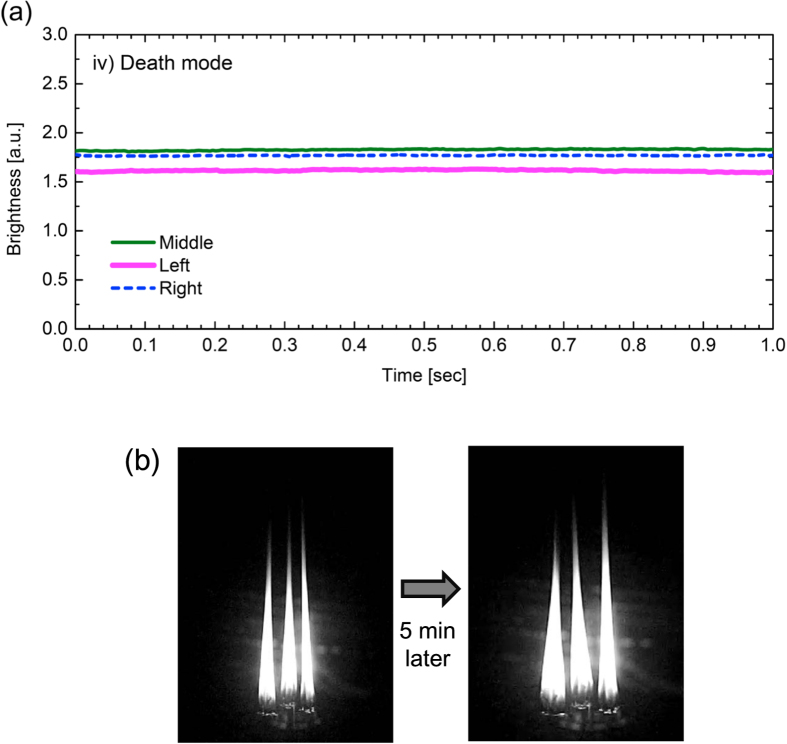
Death mode. Every flame stops oscillation and falls into self-sustained stable combustion, showing a markedly long and slender shape. (**a**) Time series of the flame brightness. (**b**) Snapshot of the oscillating flames.

The last but the most interesting synchronisation mode is the one demonstrated in Fig. 5, which we refer to as the “death” mode^16^,^17^,^18^. In the death mode, no flame oscillates; all the three flames fall into stable combustion. The observation presented in Fig. 5 was obtained by setting 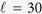 mm, while this mode is possible to occur with other different conditions of 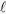; we will revisit this issue in the next section.

The death mode involves two salient features: remarkable stability and slender geometry. Firstly, the death mode is significantly robust and self-sustained, differing from the other three synchronisation modes. Once the system falls into the death mode, it persists in a duration of several minutes or more. Even when external disturbance (*e.g.*, strong air blow) is imposed to interrupt the death mode, it tends to recover after the disturbance was removed. The panel shown in Fig. 5(b) indicates the long-lived property of the death mode. After the onset of the death mode (left panel), we imposed crosswind to the flames several times during five minutes (not shown). Nevertheless, the death mode turned out to regenerate cyclically with no sign of disappearance (right panel) and persists even after that.

The second feature of the death mode is the slender shape of the combustion flame. In the death mode, the lateral width of the flame at the most bulge part (*i.e.,* just beside the wicks) was typically 7 mm under the current experimental condition. This value is significantly smaller than the lateral width of the oscillating flames. In fact, we measured the lateral width of a single oscillating flame associated with an isolated (non-coupled) bundle of three candles; the flame width fluctuated with time in the range of 10 mm–14 mm, any of which is larger than the flame width in the death mode. In addition, the vertical length of the flame in the death mode is significantly larger than those of oscillating flames. In the death mode, the typical length from the bottom of the wick to the tip of the flame is about 120 mm. This value is quite larger than the vertical length of the oscillating flames. In the case of an isolated bundle, the frame length fluctuated within the range of 70 mm–100 mm, whose maximum value is less than the typical value for the death mode. In short, the death mode provides absolutely still flames with a vertically long and laterally slender shape.

It should be emphasized that the death mode we have observed is essentially different from a mere temporal cease of the flame oscillation. In general, combustion flame often shows a transient disappearance of fluctuation in the height of the flame. However, the mere transient disappearance of fluctuation is neither endowed with the self-sustaining property against external perturbation nor the slender shape with pronounced vertical height. In this sense, the death mode we have observed is a consequence of the coupling between candle flames, so-called a limiting case of the synchronised mode with no oscillation amplitude.

## Discussion

### Statistics of synchronisation occurrence

The previous section focuses on the essential properties of four synchronised modes that the three-coupled candle flames have exhibited. It should be stressed that these four modes do not occur with equal probability; instead, which synchronised mode is preferred to occur is dependent on the inter-flame distance 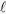. Furthermore, a growth in 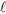 will cause a decline in the coupling strength between the oscillating flames; therefore, the rate of occurrence of synchronisation is expected to decrease with increasing 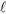.

To make clear the issue raised above, we have repeated many times the combustion experiment using different sets of three bundles, and examined the occurrence rate of synchronisation, *P*_sync_. For a fixed 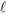, we took 12 movies (or more for 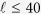 mm) with two-minute duration; from each movie, we extracted four one-second portions at random point of time, and checked whether any synchronised oscillation appears in each of the four one-second movies. This procedure allowed us to estimate *P*_sync_ over the 48 samples (or more) of one-second movies for the given 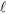. We conducted the same procedure with varying 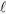 from 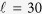 mm to 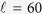 mm, and revealed the correlation between *P*_sync_ and 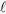.

**Figure 6:**
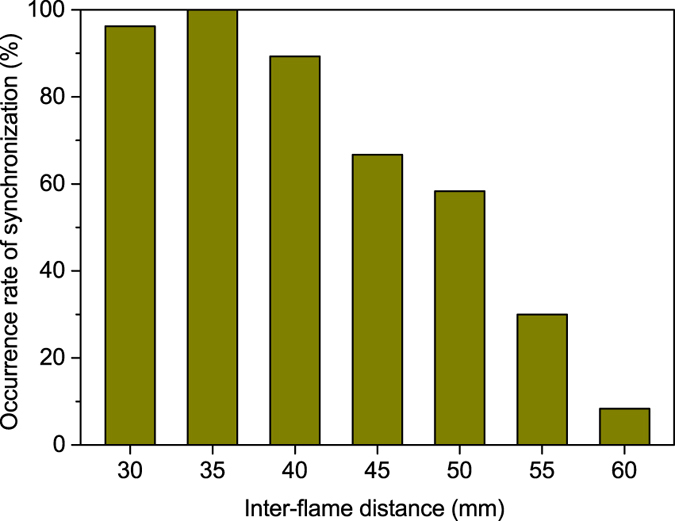
Rate of occurrence of synchronisation in three-coupled oscillating candle flames with various inter-flame distances.

Figure 6 presents the dependence of *P*_sync_ on 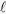 we have detected in experiments. The result clearly shows the decreasing trend of *P*_sync_ with increasing 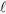 as expected. When 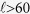 mm, the three flames were hardly coupled to each other so that they burnt independently almost anytime. It is noteworthy that the three-coupled flames with 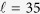 mm showed synchronisation with 100% certainty, whereas those with 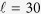 mm did not. This implies that the coupling strength between the oscillating flames is not a monotonic function of 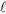; other physical or chemical factors may be responsible for the coupling strength, at least when three candle flames are more closely positioned.

**Figure 7:**
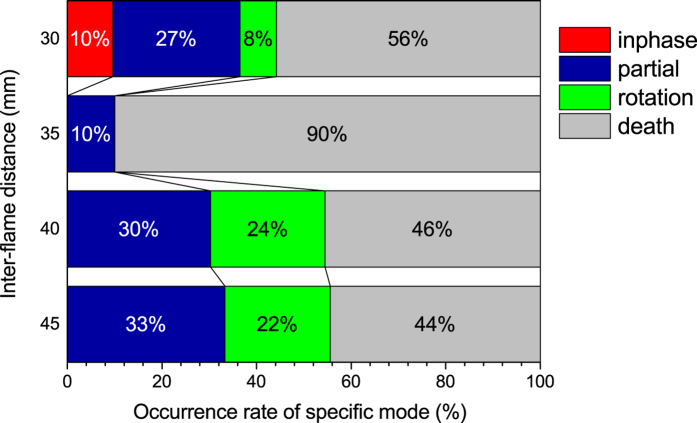
Chart showing the probability with which each synchronised mode occurs for a given inter-flame distance.

Figure 7 gives a chart that illustrates the probability with which each synchronised mode occurs for a given inter-flame distance. It is clear that the death mode occurrence occupies overall the chart at every 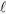, particularly at 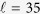 mm at which the synchronisation happens with 100% certainty under the current experimental setup. The dominancy of the death mode in the chart is thought to stem from its self-sustained property and the instability inherent to the other three modes. In addition, the remarkable high probability of the death mode occurrence at 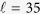 mm implies that there exists a specific condition of 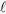 as well as the oscillating flame sizes that promotes the death mode occurrence for a given experimental apparatus.

Figure 7 also indicates that the in-phase mode rarely happens, being able to be obtained only at 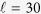 mm. This is attributed to the need of sufficiently strong coupling strength for the in-phase mode to take place, similarly to the case of paired oscillating flames^1^,^3^. We have indeed checked that a much smaller 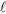 tends to increase the rate of occurrence of the in-phase mode, although not shown in the chart because of difficulty in image processing; For the smaller 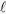, the bright areas of two adjacent flames looked overlapping in grayscale images, which made it difficult to distinguish between the two flame sizes. The Supplementary Video S1,S2,S3,S4,S5 present typical synchronised behaviours at various 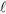
s.

### Consistency with the symmetric Hopf bifurcation theory

From a theoretical viewpoint, it would be interesting to capture the possible synchronised modes using the symmetric Hopf bifurcation theory^6^. The theory provides an elegant mathematical approach for describing pattern formation in coupled oscillator systems. The distinct feature of the theory is the model independence. In fact, in order to predict the possible synchronised mode, it suffices to know only geometrical symmetry of the system. This feature allowed to apply the theory to a wide variety of coupled oscillator systems and to obtain the list of possible synchronised patterns: gait patterns of multi-legged animals^12^, cell-shape patterns in coupled plasmodial slime molds^13^, and spatio-temporal patterns in human dynamics during sports activities^15^ are only a few to mention. It is natural to consider, therefore, whether or not our experimental results are consistent with those predicted from the symmetric Hopf bifurcation theory.

A central hypothesis of the symmetric Hopf bifurcation theory is the presence of a network of *n*-coupled identical oscillators. The *n*-coupled oscillators are assumed to be in an equilateral *n*-gonal arrangement and to be endowed with identical couplings between the oscillators. The theory states that the symmetric *n*-coupled oscillators can exhibit spatio-temporal patterns, each of which corresponds to a different isotropy subgroup of *D*_*n*_ × *S*^1^. Here, the dihedral group, *D*_*n*_, refers to the symmetries of *n*-gon, and *S*^1^ denotes the circle group that represents the translational symmetry with respect to time.

Specifically in the case of three-coupled oscillators, four synchronised patterns are predicted to take place: the in-phase pattern, the partial in-phase pattern, the rotation pattern, and the partial anti-phase pattern. Our experimental results are in fair agreement with the theoretical prediction, in the sense that the former three synchronised patterns have been observed in the three-coupled candle flame oscillation. An exception is the last one of them, the partial anti-phase pattern. In this pattern, two oscillators have identical waveforms with a phase shift by *π*, and the third oscillator has a different waveform with twice the frequency of the other two. In our experiments, however, this pattern did not take place. Another noteworthy fact is the occurrence of the death mode in the three-coupled candle flames. In view of the symmetry-based understanding, the death mode may be recognized as an extreme case of the in-phase mode, since all the three oscillators exhibit identical time sequences. In summary, we have demonstrated that the symmetric Hopf bifurcation theory is effective to provide a list of possible synchronised patterns generated by three-coupled candle flames, while the partial anti-phase mode does not appear in experiments.

### Physical mechanism of candle flame oscillation

As a final remark, we propose a conjecture on the mechanism of candle flame flickering. It should be stressed out that, despite the commonly-observed phenomena, the mechanism of candle flame flickering has not been entirely clarified. A hint for solving the problem can be taken from similar flickering phenomena observable in burner flames^19^,^20^, as previously discussed in ref. 3. In the case of burner flames, flickering is attributed to the heated jet flow from the injection nozzle and the subsequent formation of large vortices nearby the flame surface^21^. Namely, spatial inhomogeneity in the flow velocity from the flame core to the ambient still air induces an instability in the flame surface, forming toroidal vortices immediately outside the luminous flame. The vortices stretch the flame vertically and create a detached puff^22^. Periodic detachment of the puff at the flame tip followed by regrowth of the main flame from the nozzle results in a flickering appearance of the burner flame. Similar “puffing” phenomena in candle flames have been observed in ref. 3, both for paired and arrays of candles.

We speculate that the vortex-based scenario also applies to the candle flame flickering, too, as initially suggested by ref. 3. It is inferred that fusing a few small candle flames by moving the wicks to each other leads to a large flame, which produces heated jet flow in the upward direction. Then, spatial inhomogeneity in the flow velocity generates toroidal vortices nearby the flame surface, giving rise to periodic detachment of the puff from the main flame, as shown in the right panel in Fig. 3(b) and the left pabel in Fig. 4(b). This scenario is consistent with the fact that, in the candle flame experiment, fusion of several small flames triggers the flickering of the resulting large flame; namely, the strong upward flow produced by the large flame is the driving force of the vortices, leading to the periodic detachment of the puff. Unless the flame is large enough, the upward flow should be so weak that the vortices do not emerge; this is the reason why an isolated, single thin candle (not bundled) does not exhibit flickering. In short, the presence of the strong upward flow is a necessary condition for the flickering to occur. This understanding is supported by the fact that, even thin candles (not bundled) can show flame oscillation^3^ if they are close enough to each other in order for the ensemble of the flames to enhance the upward flow.

**Figure 8:**
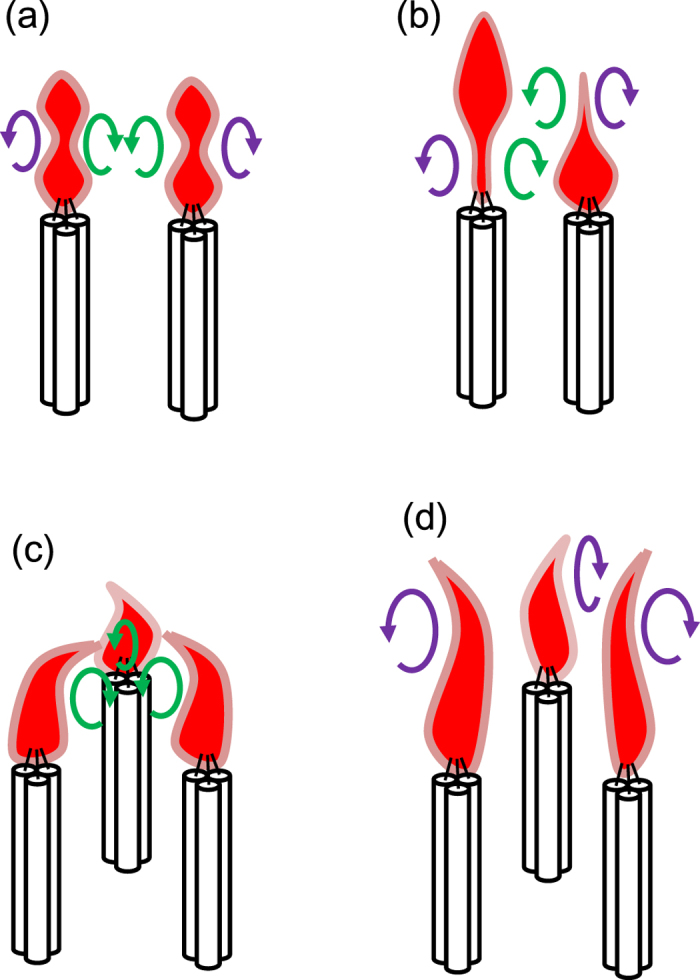
Vortex-based mechanism of the candle flame flickering. (**a**) In-phase mode of paired flames. (**b**) Anti-phase mode of paired flames. (**c**) Bowing of the three-coupled flames to the center of the triangle. (**d**) Backward arching of the three-coupled flames.

According to the vortex-based understanding, it is further inferred that the synchronised oscillation of the paired candle flames is a consequence of the interaction between the vortices and flame surfaces. A vortex generated by one flame perturbs the surface of the other flame, and vice versa; therefore, alternating perturbation between two neighbouring flames will cause the synchronisation in the coupled candle flames. Figure 8(a,b) illustrate a conceivable flickering mechanism of paired candle flames; relative configuration of the vortices determines the preferred synchronised mode, in-phase or anti-phase. We suggest that this hypothesis should hold for three-coupled candle flames, too, wherein the effect of frustration causes disappearance of the anti-phase mode. In particular, the emergence of the death mode in the three-coupled candle flames is possibly a consequence of a sudden vanishment of vortices, which tends to occur at a specific inter-flame distance as implied by our experimental finding. Once the vortices disappear, then the strong upward air flow produced by the close-set of large candle flames make the shape of the flame so sharp and self-sustained against external perturbation, as we have observed in the death mode dynamics.

The hypothesis also works well for giving an interpretation of the arching/bowing behaviour observed in the in-phase mode. Suppose that, in a given moment, the three vortices, each associated with the three oscillating flames, locate at the center of the triangular arrangement. Then the vortices repel the flame surface, and thus the three flames bow to the center; see Fig. 8(c). In contrast, when the three vortices position at the outside, the repulsive force from the vortices makes the three flame arching backward; see Fig. 8(d). Periodic alteration in the position of the vortices will lead to the periodic arching/bowing behaviour observed in the three-coupled candle flames. A further experiment on the air flow around the oscillating candle flames will give an evidence for the validity of our conjecture mentioned above.

## Conclusion

We scrutinised the synchronised oscillations of three-coupled candle flames. The system showed four types of synchronised modes: the in-phase mode, the partial in-phase mode, the rotation mode, and the death mode. The results are consistent with the theoretical prediction derived from the symmetric Hopf bifurcation theory, which provides a list of possible synchronised patterns of general coupled oscillators. Among the four, the death mode showed significant stability and self-sustaining oscillation, differing from the other three modes endowed with instability. The stability of the death mode is attributed to the vanishment of toroidal vortices nearby the flame surfaces and the strong upward air flow produced by the close-set of large candle flames.

## Additional Information

**How to cite this article**: Okamoto, K. *et al*. Synchronization in flickering of three-coupled candle flames. *Sci. Rep.* **6**, 36145; doi: 10.1038/srep36145 (2016).

**Publisher’s note:** Springer Nature remains neutral with regard to jurisdictional claims in published maps and institutional affiliations.

## Supplementary Material

Supplementary Information

Supplementary Video S1

Supplementary Video S2

Supplementary Video S3

Supplementary Video S4

Supplementary Video S5
