# Supplementary material for: Synchronization in flickering of three-coupled candle flames
Source: Sci Rep. 2016 Oct 26;6:36145. doi: 10.1038/srep36145 (PMC5080605; doi:10.1038/srep36145)
Supplement: Supplementary Information [file srep36145-s1.doc]

Supplementary information for:

“Synchronization in flickering of three-coupled candle flames”

Keiko Okamoto^1^, Akifumi Kijima^2^, Yoshitaka Umeno^3^, and Hiroyuki Shima^1*^

^1^ Department of Environmental Sciences, University of Yamanashi, 4-4-37 Takeda, Kofu, Yamanashi 400-8510 Japan

^2^ Faculty of Education, University of Yamanashi, 4-4-37 Takeda, Kofu, Yamanashi 400-8510 Japan

^3^ Institute of Industrial Science, The University of Tokyo, 4-6-1 Komaba, Meguro-ku, Tokyo 153-8505 Japan

Supplementary video legends are given below.

Video S1. Footage of an in-phase mode observed at 30mm in the inter-flame distance.

Video S2. Footage of a death mode observed at 35mm in the inter-flame distance.

Video S3. Footage of a partial in-phase mode observed at 40mm in the inter-flame distance.

Video S4. Footage of a rotation mode observed at 50mm in the inter-flame distance.

Video S5. Footage of a non-synchronized combustion observed at 80 mm in the inter-flame distance.

*To whom correspondence should be addressed: H. Shima (hshima@yamanashi.ac.jp)
